# Medical Conditions in the First Years of Life Associated with Future Diagnosis of ASD in Children

**DOI:** 10.1007/s10803-017-3130-4

**Published:** 2017-04-22

**Authors:** Stacey E. Alexeeff, Vincent Yau, Yinge Qian, Meghan Davignon, Frances Lynch, Phillip Crawford, Robert Davis, Lisa A. Croen

**Affiliations:** 1 Division of Research, Kaiser Permanente Northern California, 2000 Broadway, Oakland, CA 94612 USA; 2 McKesson Corporation, San Francisco, CA USA; 3 The Permanente Medical Group, Pediatrics, Oakland, CA USA; 4 Center for Health Research, Kaiser Permanente Northwest, Portland, OR USA; 5 Center for Biomedical Informatics, University of Tennessee Health Sciences Center, Memphis, TN USA

**Keywords:** Autism spectrum disorder, Comorbidity, Medical conditions

## Abstract

This study examines medical conditions diagnosed prior to the diagnosis of autism spectrum disorder (ASD). Using a matched case control design with 3911 ASD cases and 38,609 controls, we found that 38 out of 79 medical conditions were associated with increased ASD risk. Developmental delay, mental health, and neurology conditions had the strongest associations (ORs 2.0–23.3). Moderately strong associations were observed for nutrition, genetic, ear nose and throat, and sleep conditions (ORs 2.1–3.2). Using machine learning methods, we clustered children based on their medical conditions prior to ASD diagnosis and demonstrated ASD risk stratification. Our findings provide new evidence indicating that children with ASD have a disproportionate burden of certain medical conditions preceding ASD diagnosis.

**Electronic supplementary material:**

The online version of this article (doi:10.1007/s10803-017-3130-4) contains supplementary material, which is available to authorized users.

## Introduction

Autism spectrum disorders (ASD) are neurodevelopmental disorders defined by impaired social interaction and repetitive behaviors. The most recent estimates of ASD prevalence in the United States range from 1 to 2% (Kogan et al. 2009; CDC 2009; Blumberget al. 2013). Experienced clinicians can diagnose ASD in children as young as age 2 (Charman and Baird 2002). However, the average age of ASD diagnosis is >3 years in the general population and even older in minority populations, suggesting that many children are not diagnosed as quickly as possible (Mandell et al. 2005). Earlier diagnosis of ASD is critical because earlier treatment can reduce the degree of impairment and improve function (Lord et al. 2005). A possible first step toward earlier ASD diagnosis is risk stratification of children into groups who have lower and higher risks of ASD so that higher risk children can be monitored more closely. Clinical factors that precede an ASD diagnosis and are strongly associated with risk of ASD could be immensely useful for risk stratification.

Many children with ASD have also been diagnosed with co-occurring medical conditions during their later childhood and adulthood. The medical conditions most often reported to co-occur in children with ASD include immune conditions (Comi et al. 1999; Croen et al. 2005; Rosen et al. 2006; Sweeten et al. 2003; Vargas et al. 2005), seizure (Bauman 2010; Hara 2007; Rapin 1996; Tuchman et al. 1991; Volkmar and Nelson 1990), sleep disorders (Bauman 2010; Ferber 1996; Goldman et al. 2011; Johnson et al. 2009; Krakowiak et al. 2008; Richdale 1999), gastrointestinal disorders (Bauman 2010; Buie et al. 2010), metabolic disorders (Bauman 2010), hormone dysfunction (Bauman 2010), mitochondrial disorders (Oliveira et al. 2005; Rossignol and Frye 2012), nutritional deficits (Bauman 2010), and mood disorders (Bauman 2010). Most of these studies have focused on medical conditions that occur after the diagnosis of ASD. Much less is known about medical conditions that occur in the time prior to ASD diagnosis, i.e. the pre-diagnostic period. Furthermore, previous studies examining the time prior to ASD diagnosis rely on parent reports of child histories collected after ASD diagnosis that could be influenced by recall bias. Large-scale epidemiologic studies with prospectively collected medical data are needed to quantify meaningful and reliable estimates of the burden of co-occurring medical conditions among children with ASD during this time period before ASD diagnosis.

This study considered the diagnoses of medical conditions in the period preceding the first ASD diagnosis to achieve two objectives. First, we sought to determine whether certain medical conditions occurring in early childhood were associated with a higher risk of future ASD diagnosis. Second, we used supervised clustering methods developed for machine learning to identify risk-stratified clusters of children based on their documented medical conditions in early childhood before ASD diagnosis.

## Methods

### Study Population

The study population was drawn from the population of all children who were Kaiser Permanente (KP) members in Northern California, Georgia, and the Northwest born from January 1, 2000 through December 31, 2009. In order to capture all early medical conditions, children included in the study were required to have at least 9 months of KP membership in both their first and second years of life. Additionally, all children must have utilized health services at least once after their second birthday. We collected data from electronic medical records (EMR) and administrative claims for these children up to June 2012. KP Northern California (KPNC) is the largest and oldest group-model, pre-paid, integrated health care delivery organization in the United States, providing health care to over 3.8 million people; KP Northwest (KPNW) serves over 500,000 members in Oregon and Washington; KP Georgia (KPGA) has over 250,000 members. Table 1 shows the demographic characteristics of the study population.

**Table 1:** Demographic characteristics of the study population of 3,911 ASD cases and 38,609 matched controls, Kaiser Permanente members born January 2000 – December 2009

Demographic	Cases n (%)	Controls n (%)	p value
Sex			<0.001
Male	3198 (81.8)	19,592 (50.7)	
Female	713 (18.2)	19,015 (49.3)	
Age as of Jun 2012			0.988
2–5	1051 (26.9)	10,411 (27.0)	
6–9	1767 (45.2)	17,397 (45.0)	
10–12	1,093 (27.9)	10,801 (28.0)	
Race/ethnicity			<0.001
Asian	749 (19.1)	6544 (16.9)	
Black	282 (7.2)	2933 (7.6)	
Hispanic	825 (21.1)	9257 (24.0)	
Native American	16 (0.4)	152 (0.4)	
White	1720 (44.0)	15,801 (40.9)	
Other	319 (8.2)	3922 (10.2)	
Site			0.511
A	457 (11.7)	4334 (11.2)	
B	3274 (83.7)	32,591 (84.4)	
C	180 (4.6)	1684 (4.4)	
Median Income, census tract			<0.001
Low (≤$55,000)	1597 (41.6)	17,278 (45.7)	
Medium ($55,001–$70,000)	933 (24.3)	9539 (25.2)	
High (>$70,000)	1305 (34.0)	10,993 (29.1)	
Education past high school, census tract			0.001
Low (<53%)	1081 (28.2)	11,712 (31.0)	
Medium (53–72%)	1536 (40.0)	14,759 (39.0)	
High (>72%)	1218 (31.8)	11,336 (30.0)	

### Case and Control Definition

ASD cases were defined as children with either (a) an ASD diagnosis from an ASD specialist or (b) two or more ASD diagnoses from non-specialists, separated in time. Specialists were doctors with known expertise in pediatric ASD (many of whom were psychologists or developmental pediatricians); non-specialists were the remaining doctors. These criteria were based on an ASD case validation exercise completed across all sites (Coleman et al. 2015). Controls were required to never have had an ASD diagnosis as of June 2012. Controls were matched to cases at a 10:1 ratio on site, year and month of birth, and length of membership in the 12 months before and after the case’s first ASD diagnosis.

### Medical Conditions

We restricted our analysis to medical conditions recorded in the EMR during the “pre-diagnostic period,” defined for ASD cases as the time period prior to the first ASD diagnosis, and for matched controls as the time period prior to the age of first ASD diagnosis of the matched ASD case. Medical conditions were identified by ICD-9 codes. Over 1000 ICD-9 codes were grouped into 79 medical conditions (e.g., constipation) within 19 domains (e.g., gastrointestinal). First, based on clinician input, each condition was required to have been recorded a minimum number of times in the EMR to be considered valid (Appendix A: ESM Online). Preliminary grouping was guided by previous studies of ASD-related conditions and independently reviewed and refined by two clinicians. Additional ICD-9 codes related to each condition were identified using phenotype-wide association study methodology (Doshi-Velez et al. 2014) and reviewed by one clinician; codes deemed relevant were included for that condition. Finally, rare conditions (prevalence <0.5% among ASD cases) were regrouped into the “other” category within each domain.

## Statistical Analyses

We tabulated the count and observed prevalence of each condition among cases and among controls during the pre-diagnostic period. Conditional logistic regression analyses were carried out to estimate the odds ratio (OR) while accounting for the matched case–control design. The OR can be interpreted as a relative risk of ASD diagnosis in this study, because ASD meets the rare outcome criterion (Jewell 2003). Adjusted models accounted for the matching by site and age and adjusting for sex, maternal race, maternal education, and household income. Wald-based 95% confidence intervals (CI) were constructed for each OR using a level of 0.05. To account for multiple comparisons, we used the Bonferroni cutoff, which considers only p-values <0.00063 to be statistically significant based on testing 79 conditions.

To identify clusters of multiple conditions that were strongly associated with subsequent ASD diagnosis, we used the conditional inference tree (CIT) method (Hothorn et al. 2006). CIT is a supervised clustering method that creates clusters defined by specific combinations of conditions and detects interactions between conditions without having to pre-specify those interactions. As a supervised algorithm, the splits for each cluster are based on differentiating ASD cases from controls, resulting in a set of clusters that should distinguish condition patterns of varying association with subsequent diagnosis of ASD. To account for multiple comparisons and avoid overfitting, we used the Bonferroni cutoff for statistical significance as the stopping criteria. CIT was implemented using the party package in R (R Core Team 2014). The categorical variable created by CIT was then used as a covariate in our conditional logistic regression model, and we computed crude and adjusted ORs using the lowest risk condition cluster as the reference.

We conducted two clustering analyses of medical conditions. The first clustering analysis identified co-occurring medical condition clusters among medical conditions documented during the pre-diagnostic period. This analysis was aimed at understanding the overall burden of multiple medical conditions that occur prior to ASD diagnosis. The second clustering analysis focused specifically on medical conditions present before age 3. By focusing on conditions that occur in early life, this analysis was aimed at identifying comorbidity clusters that might have potential to improve early ASD risk stratification.

## Results

Demographic differences between cases and controls are presented in Table 1. We observed a large and significant difference in the gender of ASD cases and controls: 82% of ASD cases were males, compared with 51% of the control group. There were also small but statistically significant differences in the distributions of race/ethnicity, median income, and education. A higher percentage of ASD cases were white or Asian and a lower percentage of ASD cases were Hispanic or other compared to the control group. ASD cases also lived in areas with slightly higher median incomes and slightly higher levels of education compared to the control group. There were no statistically significant differences in the matching variables age and site. Among ASD cases, the average age of ASD diagnosis was 3.99 years.

Table 2 lists each medical condition, its prevalence among cases and among controls during the pre-diagnostic period, and the estimated odds ratio (OR) for the crude and adjusted associations with ASD risk. We found that 38 of the 79 medical conditions had statistically significant associations with ASD risk after multiple testing adjustment by the Bonferroni cutoff. Within the developmental delay domain, language delays were the most frequently diagnosed and most strongly associated with ASD risk; learning & cognitive disorders, global delays, motor delays also had strong associations. All mental health conditions were statistically significant; disruptive impulse conduct disorders, attention-deficit/hyperactivity disorders (ADHD) and anxiety disorders were the most prevalent among ASD cases during the pre-diagnostic period and had stronger associations with ASD risk than depression, adjustment disorders, tic disorder and other mental health conditions, each of which had low prevalence among ASD cases (<1%) during the pre-diagnostic period. Most neurologic conditions showed strong associations with risk of ASD. Cerebral palsy, epilepsy & recurrent seizures, and other disorders of the central nervous system had the strongest associations. Weaker but statistically significant associations were observed for other congenital anomalies of the nervous system, abnormalities of skull and face, hereditary and degenerative diseases of the central nervous system, and paroxysmal disorders.

**Table 2:** Relationship between ASD risk and individual medical conditions diagnosed in the period preceding the first ASD diagnosis

Comorbidity^a^	ASD cases N (%)	Controls N (%)	Crude model OR (95% CI)	Adjusted model OR (95% CI)^b^
Developmental delay				
Language delay	**1926 (49.25)**	**1376 (3.56)**	**26.25 (24.17–28.52)**	**23.30 (21.36–25.42)**
Learning & cognitive disorder	**21 (0.54)**	**13 (0.03)**	**16.03 (8.02–32.03)**	19.72 (9.34–41.61)^c^
Global delay	**693 (17.72)**	**476 (1.23)**	**17.25 (15.27–19.49)**	**17.16 (15.05–19.58)**
Motor delay	**164 (4.19)**	**238 (0.62)**	**7.06 (5.77–8.63)**	**7.06 (5.67–8.79)**
Mental Health				
Disruptive impulse conduct disorders	**204 (5.22)**	**120(0.31)**	**17.65 (14.05–22.17)**	**15.24 (11.96–19.42)**
Attention-deficit/hyperactivity disorders	**250 (6.39)**	**195 (0.51)**	**13.45 (11.12–16.27)**	**11.61 (9.47–14.24)**
Anxiety disorder	**125 (3.20)**	**134 (0.35)**	**9.48 (7.41–12.12)**	**10.22 (7.83–13.33)**
Depression	**23 (0.59)**	**38 (0.10)**	**6.00 (3.57–10.09)**	**5.87 (3.36–10.27)** ^c^
Adjustment disorders	**38 (0.97)**	**72 (0.19)**	**5.25 (3.54–7.79)**	**5.02 (3.31–7.59)**
Tic disorder	**25 (0.64)**	**36 (0.09)**	**6.89 (4.13–11.49)**	**4.78 (2.80–8.14)**
Other mental health	**71 (1.82)**	**122 (0.32)**	**5.83 (4.35–7.83)**	**5.74 (4.20–7.85)**
Neurology				
Cerebral palsy	**45 (1.15)**	**106 (0.27)**	**4.23 (2.98-6.00)**	**4.92 (3.38–7.14)**
Other disorders of central nervous system	**1454 (37.18)**	**5725 (14.83)**	**3.40 (3.17–3.65)**	**3.39 (3.15–3.65)**
Epilepsy & recurrent seizures	**194 (4.96)**	**610 (1.58)**	**3.25 (2.76–3.83)**	**3.38 (2.84–4.02)**
Other congenital anomalies of nervous system	**80 (2.05)**	**299 (0.77)**	**2.68 (2.09–3.43)**	**2.79 (2.15–3.61)**
Abnormalities of Skull and Face	**141 (3.61)**	**510 (1.32)**	**2.79 (2.31–3.38)**	**2.56 (2.10–3.12)**
Hereditary and degenerative diseases of central nervous system	**56 (1.43)**	**248 (0.64)**	**2.25 (1.68–3.01)**	**2.19 (1.61–2.96)**
Paroxysmal	**221 (5.65)**	**1122 (2.91)**	**2.00 (1.73–2.32)**	**2.01 (1.73–2.35)**
Disorders of peripheral nervous system	27 (0.69)	154 (0.40)	1.74 (1.15–2.62)	1.66 (1.08–2.54)
Headache	20 (0.51)	154 (0.40)	1.28 (0.80–2.05)	1.24 (0.76–2.02)
Other neurology	46 (1.18)	263 (0.68)	1.74 (1.27–2.38)	1.68 (1.20–2.35)
Nutrition				
Symptom concerning nutrition metabolism and development	**1517 (38.79)**	**6429 (16.65)**	**3.17 (2.96–3.40)**	**3.18 (2.95–3.42)**
Genetic conditions	**39 (1.00)**	**133 (0.34)**	**2.91 (2.04–4.17)**	**3.13 (2.15–4.54)**
Ear, nose and throat conditions	**711 (18.18)**	**3097 (8.02)**	**2.55 (2.33–2.79)**	**2.47 (2.25–2.72)**
Sleep				
Organic sleep apnea	**47 (1.20)**	**195 (0.51)**	**2.40 (1.74–3.30)**	**2.17 (1.55–3.03)**
Dyssomnia	**189 (4.83)**	**851 (2.20)**	**2.25 (1.92–2.65)**	**2.11 (1.78–2.49)**
Gastrointestinal conditions				
GERD	**262 (6.70)**	**1163 (3.01)**	**2.31 (2.01–2.65)**	**2.20 (1.90–2.55)**
Diarrhea	**215 (5.50)**	**1217 (3.15)**	**1.79 (1.54–2.07)**	**1.72 (1.48–2.01)**
Constipation	**323 (8.26)**	**2225 (5.76)**	**1.47 (1.30–1.66)**	**1.62 (1.43–1.84)**
Upper GI diseases	**113 (2.89)**	**596 (1.54)**	**1.90 (1.55–2.33)**	**1.61 (1.30–1.99)**
Upper GI motility	**269 (6.88)**	**1721 (4.46)**	**1.58 (1.39–1.81)**	**1.54 (1.34–1.77)**
Functional disorders	188 (4.81)	1658 (4.29)	1.13 (0.96–1.31)	1.16 (0.99–1.36)
Lower GI	71 (1.82)	645 (1.67)	1.09 (0.85–1.39)	1.00 (0.77–1.29)
Other disease of esophagus	**47(1.20)**	**225 (0.58)**	**2.08 (1.51–2.85)**	**2.10 (1.51–2.91)**
Other GI	**627 (16.03)**	**4727 (12.24)**	**1.37 (1.25–1.50)**	**1.37 (1.24–1.51)**
Metabolic				
Fluid, electrolyte, and acid-base balance	**106 (2.71)**	**584 (1.51)**	**1.81 (1.47–2.24)**	**1.78 (1.43–2.21)**
Overweight, obesity and other hyperalimentation	**102 (2.61)**	**640 (1.66)**	**1.59 (1.29–1.96)**	**1.63 (1.31–2.04)**
Musculoskeletal	**562 (14.37)**	**3354 (8.69)**	**1.76 (1.60–1.94)**	**1.69 (1.53–1.86)**
Ophthalmology	**676 (17.28)**	**4239 (10.98)**	**1.69 (1.55–1.85)**	**1.66 (1.52–1.83)**
Pulmonary	**270 (6.90)**	**1970 (5.10)**	**1.38 (1.21–1.57)**	**1.28 (1.11–1.46)**
Autoimmune				
Inflammatory bowel disease	**603 (15.42)**	**4618 (11.96)**	**1.34 (1.22–1.47)**	**1.34 (1.22–1.48)**
Other autoimmune	57 (1.46)	374 (0.97)	1.51 (1.14-2.00)	1.54 (1.15–2.05)
Other autoimmune dermatitis	25 (0.64)	186 (0.48)	1.33 (0.87–2.02)	1.40 (0.90–2.16)
Infections				
Bacterial	241 (6.16)	2262 (5.86)	1.06 (0.92–1.21)	1.06 (0.92–1.22)
Viral	2338 (59.78)	21,789 (56.44)	1.15 (1.07–1.23)	1.10 (1.02–1.18)
Ear	2770 (70.83)	26,381 (68.33)	1.13 (1.05–1.21)	1.07 (0.99–1.15)
Eye	1536 (39.27)	15,226 (39.44)	0.99 (0.93–1.06)	0.94 (0.88–1.01)
Gastrointestinal	**819 (20.94)**	**6908 (17.89)**	**1.22 (1.12–1.32)**	**1.19 (1.09–1.29)**
Female genitourinary	46 (1.18)	1253 (3.25)	0.35 (0.26–0.48)	0.97 (0.71–1.32)
Genitourinary	130 (3.32)	1847 (4.78)	0.68 (0.57–0.82)	1.09 (0.90–1.31)
Lower respiratory	**1290 (32.98)**	**13,177 (34.13)**	**0.95 (0.89–1.02)**	**0.87 (0.81–0.94)**
Upper respiratory	3430 (87.70)	33,734 (87.37)	1.03 (0.93–1.14)	0.95 (0.85–1.05)
Lymph	146 (3.73)	1303(3.37)	1.11 (0.93–1.32)	1.00 (0.84–1.20)
Mycoses	939 (24.01)	9165 (23.74)	1.02 (0.94–1.10)	1.10 (1.02–1.20)
Skin	754 (19.28)	7583 (19.64)	0.98 (0.90–1.06)	0.95 (0.87–1.04)
Other infection	199 (5.09)	1494 (3.87)	1.33 (1.14–1.55)	1.26 (1.08–1.48)
Hematology				
Hematology anemia	147 (3.76)	1135 (2.94)	1.29 (1.08–1.54)	1.27 (1.06–1.52)
Neoplasms	108 (2.76)	924 (2.39)	1.16 (0.95–1.42)	1.25 (1.01–1.54)
Other hematology	36 (0.92)	262 (0.68)	1.36 (0.96–1.93)	1.31 (0.91–1.87)
Allergy				
Allergic rhinitis	266 (6.80)	2101 (5.44)	1.27 (1.11–1.45)	1.15 (1.00-1.32)
Atopic dermatitis	825 (21.09)	7279 (18.85)	1.15 (1.06–1.25)	1.10 (1.01–1.20)
Conjunctivitis	761 (19.46)	6850 (17.74)	1.12 (1.03–1.22)	1.03 (0.94–1.12)
Contact dermatitis and eczema	838 (21.43)	7729 (20.02)	1.09 (1.01–1.18)	1.07 (0.98–1.16)
Drug allergy	108 (2.76)	805 (2.09)	1.33 (1.09–1.63)	1.29 (1.04–1.59)
Food allergy	112 (2.86)	683 (1.77)	1.64 (1.34-2.00)	1.37 (1.11–1.69)
Serious local reactions	304 (7.77)	3090 (8.00)	0.97 (0.86–1.10)	0.94 (0.83–1.06)
Stevens-Johnson syndrome	23 (0.59)	159 (0.41)	1.43 (0.92–2.22)	1.25 (0.80–1.95)
Urticaria	277 (7.08)	2388 (6.19)	1.16 (1.02–1.32)	1.08 (0.95–1.24)
Other allergies	398 (10.18)	3446 (8.93)	1.16 (1.04–1.29)	1.07 (0.95–1.19)
Genitourinary				
Genital tract	166 (4.24)	893 (2.31)	1.87 (1.58–2.22)	1.13 (0.95–1.34)
Renal disorders	112 (2.86)	1231 (3.19)	0.90 (0.74–1.09)	1.18 (0.97–1.45)
Injury				
Accidental poisoning	1461 (37.36)	14,032 (36.34)	1.04 (0.98–1.12)	1.12 (1.02–1.23)
Fracture	239 (6.11)	2407 (6.23)	0.98 (0.85–1.12)	0.92 (0.80–1.07)
Injury of head or neck	456 (11.66)	3570 (9.25)	1.30 (1.17–1.44)	1.19 (1.07–1.32)
Cardiovascular disorders	30 (0.77)	122 (0.32)	2.44 (1.63–3.64)	2.02 (1.30–3.13)
Endocrine disorders	30 (0.77)	218 (0.56)	1.36 (0.93-2.00)	1.86 (1.25–2.77)
Other				
Fever of unknown	385 (9.84)	3548 (9.19)	1.08 (0.97–1.21)	1.05 (0.93–1.20)
Unspecified adverse effect of drug	94 (2.40)	737 (1.91)	1.27 (1.02–1.57)	1.23 (0.98–1.54)
Asthma	1014 (25.93)	9564 (24.77)	1.06 (0.99–1.15)	0.94 (0.86–1.01)

^a^Results are listed in the order of the condition domains with the strongest associations. Conditions in bold indicate statistical significance of the adjusted OR at the strict Bonferroni level accounting for multiple testing across all conditions

*OR* Odds ratio, *CI* confidence interval

^b^The adjusted logistic regression model included matching by site and age and adjusting by sex, maternal race, and census tract levels of education and household income

^c^This regression model was not convergent

Conditions in the domains of nutrition; genetics; sleep; and ear, nose & throat demonstrated moderate increases in ASD risk. Statistically significant increases in ASD risk of low to moderate magnitude were observed for conditions in the domains of ophthalmology, pulmonary, metabolic, musculoskeletal, and gastrointestinal (GI) conditions. Conditions in the domains of autoimmune, infections, hematology, allergy, injury, cardiovascular, endocrine, and asthma had weak associations, most of which were not strong enough to reach our Bonferroni cutoff for statistical significance.

### Associations of Condition Clusters Using Supervised Clustering

Table 3 shows the results from our first clustering analysis with condition clusters selected by conditional inference tree regression. The reference cluster (children without developmental delay; mental health; ear, nose & throat; nutrition; or ophthalmology) has the lowest risk of ASD diagnosis and includes 66.81% of the controls and 19.66% of the ASD cases. The condition clusters are listed from lowest to highest OR in comparison to the reference group, illustrating the increasing relative risk of ASD diagnosis. Note that the clusters are mutually exclusive such that every child belongs to only one cluster, in contrast to the results displayed in Table 2 where conditions are studied individually.

**Table 3:** Associations of child medical condition clusters with risk of ASD up to age 12, comparing medical conditions diagnosed before the first ASD diagnosis to medical conditions diagnosed during the same time period among matched controls

Cluster description^a^	ASD cases N (%)	Controls N (%)	Crude model OR (95% CI)	Adjusted model OR (95% CI)^b^	Latency time in months, Mean (SD)^c^
**No mental health or developmental delay diagnosis**					
No diagnoses of ear, nose & throat, nutrition, or ophthalmology	769 (19.66	25,796 (66.81)	Reference	Reference	No comorbidities
Ophthalmology, but no diagnoses of ear, nose & throat or nutrition	134 (3.43)	2850 (7.38)	1.57 (1.29–1.90)	1.50 (1.24–1.83)	35.9 (21.9)
Ear, nose & throat diagnosis, but no nutrition diagnosis	116 (2.97)	2062 (5.34)	1.98 (1.61–2.43)	1.93 (1.56–2.38)	20.9 (18.1)
Nutrition diagnosis, but no ear, nose & throat diagnosis	308 (7.88)	5004 (12.96)	2.23 (1.94–2.56)	2.19 (1.90–2.52)	22.3 (21.8)
Ear, nose & throat dx and nutrition diagnoses	61 (1.56)	554 (1.43)	4.29 (3.22–5.71)	4.21 (3.14–5.66)	29.8 (25.7)

**Mental health (MH) diagnosis but no developmental delay**					
MH diagnosis other than attention-deficit/hyperactivity disorder or disruptive impulse conduct disorder	65 (1.66)	269 (0.70)	9.11 (6.73–12.31)	9.12 (6.68–12.46)	14.1 (17.8)
Attention-deficit/hyperactivity disorder, but no disruptive impulse conduct disorder	99 (2.53)	128 (0.33)	29.26 (21.53–39.78)	23.10 (16.83–31.72)	14.8 (15.5)
Disruptive impulse conduct disorder	106 (2.71)	102 (0.26)	44.29 (32.38–60.58)	38.32 (27.55–53.31)	15.2 (16.6)

**Developmental delay**					
Motor delay but no language delay	64 (1.64)	181 (0.47)	11.56 (8.42–15.88)	10.56 (7.52–14.84)	20.6 (16.0)
Global delay or delay in cognitive or learning, but no language or motor delay	26 (6.72)	287 (0.74)	31.67 (25.88–38.75)	29.97 (24.24–37.07)	15.2 (19.9)
Language delay, but no dx of global delay, MH, or other disorders of central nervous system	912 (23.32)	883 (2.29)	34.80 (30.56–39.64)	28.82 (25.18–32.99)	12.9 (14.4)
Language delay and other disorders of central nervous system, but no global delay or MH diagnosis	472 (12.07)	309 (0.80)	51.90 (43.45–61.99)	42.84 (35.59–51.55)	15.4 (15.7)
Language delay and MH, but no global delay	133 (3.40)	46 (0.12)	103.67 (70.96–151.46)	78.54 (53.46–115.39)	32.7 (25.5)
Language delay and global delay	409 (10.46)	138 (0.36)	104.64 (83.37–131.34)	101.71 (79.92–129.44)	14.9 (15.7)

^a^Condition clusters selected by conditional inference tree regression identifying combinations of conditions along the main branches (in bold) of whether a developmental delay or mental health condition had been diagnosed

^b^The adjusted conditional logistic regression model includes matching by site and age and adjusting by sex, maternal race, and census tract levels of education and household income

^c^Latency time between having all conditions in cluster and being diagnosed with ASD among ASD cases

Developmental delay and mental health condition clusters were associated with the highest relative risks of ASD. Children with language delay were categorized into four distinct condition clusters; the relative risks for ASD were very high in each cluster but also showed substantial variability (ORs ranged from 28 to 101). The cluster of language delay with global delay had the highest relative risk. Among children without developmental delays, three mental health condition clusters emerged with 9–38-fold elevated risks of ASD compared to children in the lowest risk category. The disruptive impulse conduct disorder cluster had the highest relative risk for ASD and the next highest relative risk cluster included children with ADHD but without disruptive impulse conduct disorder. Beyond the mental health and developmental delay domains, condition clusters with moderate to low increases in ASD risk were defined by combinations of ophthalmology; ear, nose & throat; and nutrition conditions.

Table 4 displays the results from our second clustering analysis focused on early ASD predictors by further restricting the pre-diagnostic period conditions to those identified prior to age 3. The reference cluster (children without developmental delay; ear, nose & throat; nutrition; ophthalmology; musculoskeletal; or constipation) has the lowest risk of ASD diagnosis and includes 65.31% of the controls and 29.30% of the ASD cases. Compared to the reference cluster, developmental delay condition clusters have the strongest associations with ASD risk. The three clusters with the highest relative risks of ASD all included language delay, with substantial heterogeneity in the relative risk depending on the co-occurrence of global delay or other disorders of the central nervous system. The mental health conditions do not appear in any cluster definitions. Outside of the developmental delay domain, the conditions of seizures; musculoskeletal; ear, nose & throat; nutrition; ophthalmology and gastrointestinal conditions defined eight condition clusters, each associated with low to moderate increases in ASD risk.

**Table 4:** Associations of early life child medical condition clusters with risk of ASD up to age 12, comparing medical conditions diagnosed before age 3 and before the first ASD diagnosis to medical conditions diagnosed during the same time period among matched controls

Cluster description^a^	ASD cases N (%)	Controls N (%)	Crude model OR (95% CI)	Adjusted model^b^ OR (95% CI)	Latency Time in months mean (SD)^c^
**No developmental delay diagnosis**					
No diagnoses of ear, nose & throat, nutrition, ophthalmology, musculoskeletal, or constipation	1146 (29.30)	25,216 (65.31)	Reference	Reference	No comorbidities
Constipation, but no dx of gastroesophageal reflux disease, ear, nose & throat, nutrition, ophthalmology, musculoskeletal	66 (1.69)	1067 (2.76)	1.35 (1.04–1.75)	1.46 (1.12–1.90)	46.5 (27.2)
Musculoskeletal, but no diagnoses of ear, nose & throat, nutrition, or ophthalmology	121 (3.09)	1,783 (4.62)	1.55 (1.27–1.89)	1.45 (1.19–1.78)	43.7 (27.1)
Ophthalmology, but no diagnoses of nutrition or ear, nose & throat	191 (4.88)	2620 (6.79)	1.63 (1.38–1.91)	1.59 (1.34–1.87)	50.3 (27.0)
Ear, nose & throat, but no diagnoses of nutrition, or ophthalmology	105 (2.68)	1340 (3.47)	1.83 (1.48–2.26)	1.75 (1.41–2.18)	31.2 (26.4)
Nutrition diagnosis but no seizures	395 (10.10)	4713 (12.21)	1.99 (1.76–2.24)	1.99 (1.75–2.25)	36.3 (30.3)
Ear, nose & throat and ophthalmology, but no nutrition diagnosis	26 (0.66)	149 (0.39)	4.09 (2.66–6.30)	3.73 (2.38–5.87)	35.9 (34.1)
Constipation and gastroesophageal reflux disease, but no diagnoses of ear, nose & throat, nutrition, ophthalmology, musculoskeletal	13 (0.33)	55 (0.14)	5.09 (2.71–9.56)	5.15 (2.66–9.97)	39.5 (20.1)
Nutrition and seizure diagnoses	17 (0.43)	73 (0.19)	5.50 (3.16–9.57)	5.63 (3.13–10.15)	40.4 (25.2)

**Developmental delay diagnosis**					
Motor delay but no language delay	41 (1.05)	167 (0.43)	5.23 (3.64–7.50)	4.75 (3.20–7.05)	24.4 (18.0)
Global delay or delay in cognitive or learning, but no language or motor delay	208 (5.32)	273 (0.71)	18.15 (14.80-22.27)	17.15 (13.81–21.29)	19.9 (24.2)
Language delay, but no dx of global delay, or other disorders of central nervous system	909 (23.24)	810 (2.10)	25.26 (22.38–28.51)	20.70 (18.28–23.48)	16.5 (17.2)
Language delay and other disorders of central nervous system, but no global delay	399 (10.20)	239 (0.62)	36.08 (30.09–43.26)	29.81 (24.64–36.06)	14.4 (16.9)
Language delay and global delay	274 (7.01)	104 (0.27)	62.60 (48.71–80.45)	60.82 (46.67–79.25)	15.6 (16.1)

^a^Condition clusters selected by conditional inference tree regression identifying combinations of conditions along the main branches (in bold) of whether a developmental delay had been diagnosed

^b^The adjusted conditional logistic regression model includes matching by site and age and adjusting by sex, maternal race, and census tract levels of education and household income

^c^Latency time between having all conditions in cluster and being diagnosed with ASD among ASD cases

Latency time in months between diagnosis of all conditions in the cluster and subsequent diagnosis of ASD is reported in the last column of Tables 3 and 4, computed only among ASD cases. When a cluster includes multiple conditions diagnosed on different dates, the diagnosis date for the last condition identified is used, since diagnosis of all conditions are required to be identified in that cluster. We found that the mean latency time was greater than 12 months for every cluster identified in Tables 3 and 4, with mean latency time as high as 50 months. In general, condition clusters involving developmental delays or mental health conditions had shorter latency times than other condition clusters; however, the cluster of children diagnosed with a language delay and a mental health condition who had no diagnosed global delay had the longest mean latency time in Table 3 (mean = 32.7 months, SD = 25.5).

## Discussion

By leveraging comprehensive prospectively recorded EMR data on a large and diverse population of children in an integrated health care delivery system, this study generated robust estimates of the burden of childhood medical conditions diagnosed early in life among children subsequently diagnosed with ASD. Our manuscript presents three key findings. First, our study provides new evidence indicating that children who are diagnosed with ASD experience a higher burden of 38 medical conditions in the years preceding ASD diagnosis, including GI conditions, developmental delays, mental health disorders, musculoskeletal abnormalities, neurological conditions, and sleep disorders. Second, our highly-powered study found no evidence that children who are diagnosed with ASD experience a higher burden of the other 41 medical conditions in the years preceding ASD diagnosis, even though the conditions were all hypothesized to be more common in children with ASD based on previous studies. Third, we demonstrated how combinations of medical comorbidities could aid in risk stratification for ASD prior to ASD diagnosis using a supervised clustering analysis based on machine-learning methods. We identified 14 risk clusters such that ASD risk increases across these strata, with ORs ranging from 1.5 to 100.

### Medical Comorbidities Documented Before ASD Diagnosis

The associations of 38 medical conditions with future ASD diagnosis reported here strengthen the evidence presented in previous studies of the higher prevalence of gastrointestinal conditions, developmental delays, mental health disorders, musculoskeletal abnormalities, neurological conditions, and sleep disorders among children with ASD. In contrast to the few previous studies that relied on parent reports of medical conditions in the years before ASD diagnosis—which is highly subject to recall bias—the key innovation of our study is that we relied on diagnoses made by clinicians and recorded in the child’s medical record prospectively before the ASD diagnosis was made. Our findings provide new evidence indicating that children with ASD have a disproportionate burden of certain medical conditions in the years preceding ASD diagnosis.

A high prevalence of GI conditions among children with ASD has been reported in several studies with no comparison to control children (Molloy and Manning-Courtney 2003; Nikolov et al. 2009; Ming et al. 2008). However, a recent consensus report evaluating the diagnosis and treatment of GI conditions among individuals with ASD concluded that: “The prevalence of gastrointestinal abnormalities in individuals with ASDs is incompletely understood,” and went on to highlight the methodological limitations of previous studies and the possible influence of referral bias (Buie et al. 2010). For example, one recent study using descriptive reports from structured interviews estimated the prevalence of GI symptoms among children with ASD to be 70% compared to a prevalence of only 28% among control children (Valicenti-McDermott et al. 2006). In stark contrast, a small retrospective case–control study of the medical records of 96 ASD children and 449 control children reported that children with ASD did not have higher odds of a history of GI disorders before ASD diagnosis compared to children without ASD (OR 1.0, 95% CI 0.5–2.2) (Black et al. 2002). This study is the most similar in design to ours, although much smaller, and the CI bounds are consistent with our results (adjusted OR estimates ranging from 1.0 to 2.2 across GI conditions). Overall, our findings support our hypothesis that children with ASD are more likely than control children to have a documented comorbid GI condition in the time before ASD diagnosis, but the difference is quite modest, with effects smaller than we expected. Our findings suggest that descriptive reporting of gastrointestinal conditions may substantially over-estimate the difference in prevalence between children with ASD and controls.

We found that developmental delays and mental health conditions showed the strongest associations with future ASD diagnosis. The strong association between language delay and ASD risk is not surprising given that language delay is a component that characterizes ASD diagnosis (Rapin 1991; American Psychiatric Association 2013). Notably, we found a moderately strong association of motor delay with future ASD diagnosis. Motor delays are not considered prodromal to ASD development, but some studies have reported increased motor impairment among children already diagnosed with ASD as compared to control children (Noterdaeme et al. 2002; Mari et al. 2003). To our knowledge, our finding of an association between ASD and a motor delay diagnosis that precedes ASD diagnosis has not previously been reported.

For 41 other medical conditions, we did not find statistically significant evidence of an association with ASD risk in this highly-powered study. We found that children who are later diagnosed with ASD were not more likely than controls to be diagnosed with allergy, asthma, infections, autoimmune conditions, hematology, genitourinary, accidental injury, endocrine disorders or cardiovascular disorders in the years preceding ASD diagnosis. In contrast, some previous studies have reported that ASD is associated with comorbid medical conditions of allergies (Yaghmaie et al. 2013; Theije et al. 2014), asthma (Kotey et al. 2014), infections (Atladottir et al. 2010), autoimmune conditions (Gesundheit et al. 2013), and endocrine disorders (Chen et al. 2009).

We previously reported that both allergies and autoimmune diseases were diagnosed more often among children already diagnosed with autism than among controls (Zerbo et al. 2015). One possible explanation for why results during the time period prior to ASD diagnosis differ from results when studying children already diagnosed with ASD is that some medical conditions may have a later age of onset than the age of ASD diagnosis. Alternatively, increased health care utilization among children already diagnosed with ASD could lead to increased opportunity for diagnoses of other medical conditions relative to children without ASD who have fewer encounters with the health care system. Further research is needed to elucidate how medical conditions that co-occur with ASD may differ before and after ASD diagnosis and the reasons driving those differences.

### ASD Risk Stratification Based on Medical Comorbidities

Our study also demonstrated the ability of medical condition clusters to differentiate relative risks of ASD. We identified 14 risk strata and illustrated a gradient of increasing ASD risk across these strata, with ORs ranging from 1.5 to 100 in comparison to the lowest risk group of children. Risk stratification of children into groups who have lower and higher risks of ASD could be a first step toward earlier ASD diagnosis by identifying groups of children for whom increased monitoring or earlier screening protocols could be developed. Earlier diagnosis of ASD is critical because earlier treatment can reduce the degree of impairment and improve function (Lord et al. 2005). Our analysis of the latency time between diagnosis of all conditions in each cluster and subsequent diagnosis of ASD found that the mean latency time was greater than 12 months for every cluster, and as high as 50 months for some, illustrating that there is a substantial window of time between identification of higher risk children and the diagnosis of ASD. Not surprisingly, the high risk clusters were dominated by developmental delays and mental health conditions. However, there were interesting features regarding how these delays clustered together in different combinations. Interestingly, both cluster analyses identified several distinct clusters within the high risk group of children with language delays, where language delays in combination with certain other comorbid conditions showed heterogeneous relative risks for ASD that were all higher than the relative risk in the cluster of language delay alone. These results underscore the importance of considering *combinations* of conditions. Global delay sometimes incorporates language delay because global developmental delay is defined as significant delay in two or more areas of development (American Psychiatric Association 2013). Children diagnosed with a global delay who do not have a separate language delay diagnosis may have a language delay as a component. Thus, it is difficult to interpret the higher relative risk for the cluster of global delay with language delay compared to the cluster of global delay without language delay. Our findings of an association between motor delay and ASD are consistent with other recent studies suggesting an increased prevalence of motor impairment among those with ASD as compared to controls (Ming et al. 2007). Notably, our cluster analyses showed an association of motor delay with ASD risk even among children who had no co-occurring language delay diagnosis.

Mental health conditions in the pre-diagnostic period showed very strong associations with subsequent ASD diagnosis in the main analysis. The all-age cluster analysis showed that mental health diagnoses were important in both the absence and the presence of developmental delays. These mental health condition clusters were not identified in the age 3 cluster analysis which was restricted to medical conditions identified before age 3. This finding is not surprising given that typical age-of-onset is 4–11 years for ADHD and 5–15 years for conduct disorders (Kessler et al. 2007). Increased monitoring or screening of children with mental health conditions may be important considering the evidence of (i) the strength and timing of these associations with mental health conditions, (ii) more than 10% of ASD cases were diagnosed with a mental health condition prior to their first ASD diagnosis, and (iii) the cluster of children diagnosed with a language delay and a mental health condition who had no diagnosed global delay had the longest mean latency time in Table 3 (mean = 32.7 months, SD = 25.5). These findings are supported by a recent study of children with ASD, ages 2–17, in which approximately 20% of ASD cases were first diagnosed with ADHD, and on average those children were not diagnosed with ASD until 3 years after their ADHD diagnosis (Miodovnik et al. 2015).

Outside of the domains of mental health and developmental delay, the conditions of ophthalmology; ear, nose and throat; and nutrition defined other condition clusters. Individually, each condition was also associated with a moderate increase in ASD risk. The conditions of constipation, gastroesophageal reflux disease, musculoskeletal abnormalities, and seizures contributed to defining the clusters only when studying conditions diagnosed before age 3. Several conditions reached Bonferroni significance in individual analyses but not cluster analyses, including metabolic, gastrointestinal, genetic, pulmonary, sleep, and neurologic conditions; these conditions tended to have either low prevalence or a weak association with ASD risk (OR < 2).

Several recent studies of ASD have utilized a clustering approach to identify risk factors, but the focus of those studies differs from ours. Doshi-Velez et al. only examined children diagnosed with ASD and sought to cluster underlying patterns that may represent heterogeneous etiologic pathways for ASD (Doshi-Velez et al. 2014). Brennan et al. focused on symptoms identified in screening protocols and used hierarchical cluster analyses to detect underlying clinically distinct subgroups (Brennan et al. 2015). Chawarska et al. studied younger siblings of children with ASD, a high risk population, and used a tree-based method to identify clusters of behavioral features associated with subsequent ASD diagnosis or atypical development (Chawarska et al. 2014). In contrast, our study included children with ASD and age-matched controls and identified condition clusters that indicated increased risk of ASD in the pre-diagnostic period.

### Strengths and Limitations

Our study has a number of strengths and limitations. Our large and diverse study population across three health systems in different parts of the U.S. contributes to the generalizability of these results. Physician documentation of conditions was recorded prospectively across a broad range of medical conditions prior to ASD diagnosis, preventing recall bias and diagnostic bias. However, the ascertainment of medical diagnoses relies on the documentation in each record, so differential utilization of health services that leads to a differential reporting of conditions by case–control status could bias our results. In addition, there is potential for some misclassification in ASD phenotypic assessment since not all diagnoses were made by experts.

A statistical strength of this study is the Bonferroni control for multiple testing in individual and cluster analyses. A concern in tree-based supervised clustering is model overfitting by creating too many clusters that are not meaningful and cannot be replicated (Strobl et al. 2009). Pruning, a cross-validation procedure, can combat overfitting, but a strength of our study is using a Bonferroni test for the stopping criteria which provides a strong degree of control for overfitting without loss of predictive performance (Hothorn et al. 2006). The main statistical limitation of our analyses is that the matched case–control design prohibits us from calculating an estimate and confidence interval for the absolute risk, thus limiting our statistical inference to ORs (Agresti and Kateri 2011). We cannot directly evaluate the predictive characteristics of this clustering model in terms of the absolute risk of ASD for each cluster; this is a topic for future work. This future work could extend to quantifying the sensitivity and specificity of different screening protocols that might be targeted to particular high risk clusters. Although we have demonstrated risk stratification with a gradient of increasing relative risk across clusters, the clinical utility of integrating the risk stratification with screening protocols would depend on a number of factors including the strength of the association of each risk cluster with ASD and the proportions of cases and controls in each risk cluster.

## Conclusion

By taking advantage of the comprehensive EMR data available for a large and diverse population of children in an integrated health care delivery system, this study has generated robust estimates of the burden of medical conditions among children with ASD during the pre-diagnostic period. This work can increase awareness of the diversity of conditions affecting children with ASD prior to their first ASD diagnosis. Further study of conditions during the pre-diagnostic period may increase our ability to identify children at risk of having ASD, and improved risk stratification may lead to earlier diagnoses and interventions.

## Electronic supplementary material

Below is the link to the electronic supplementary material.

Supplementary material 1 (DOCX 47 KB)

## Abbreviations

ASD: Autism spectrum disorders
ADHD: Attention-deficit/hyperactivity disorders
GI: Gastrointestinal
EMR: Electronic medical records
KP: Kaiser Permanente
KPGA: Kaiser Permanente Georgia
KPNW: Kaiser Permanente Northwest
KPNC: Kaiser Permanente Northern California
OR: Odds ratio
