# Supplementary material for: Medical Conditions in the First Years of Life Associated with Future Diagnosis of ASD in Children
Source: J Autism Dev Disord. 2017 Apr 22;47(7):2067–79. doi: 10.1007/s10803-017-3130-4 (PMC5487747; doi:10.1007/s10803-017-3130-4)
Supplement: Supplementary file 1 — Supplementary material 1 (DOCX 47 KB) [file 10803_2017_3130_MOESM1_ESM.docx]

Appendix A: ICD-9 Codes and Comorbidity Classifications

| **Allergy** | **ICD-9 Codes** | **Number of Required Diagnoses** |
| --- | --- | --- |
| Allergic Rhinitis | 477.xx | 2 or more diagnoses within same category |
| Atopic Dermatitis | 691.xx | 2 or more diagnoses within same category |
| Conjunctivitis | 372.10x 372.11x 372.12x 372.13x 372.14x 372.2x 372.3x 372.0x | 2 or more diagnoses within same category |
| Contact dermatitis and eczema | 692.0x 692.1x 692.4x 692.6x 692.70x 692.71x 692.72x 692.76x 692.79x 692.81x 692.83x 692.89x 692.9x 692.5x 373.32x | 1 or more diagnoses with same ICD-9 Code |
| Drug allergy | 692.3x 999.42x V14.0 V14.1 V14.2 V14.3 V14.4 V14.5 V14.6 V14.7 V14.8 V14.9 995.2x V64.04 995.4x | 1 or more diagnoses with same ICD-9 Code |
| Food allergy | V15.01 V15.02 693.1x 995.6x V15.03 V15.04 V15.05 | 1 or more diagnoses with same ICD-9 Code |
| Other allergies | 287.0x 381.04x 381.05x 381.06x 999.4x V15.0x ,995.3x 989.82x V15.07 995.0x; 995.6x, 999.4x 995.1x 780.63x | 1 or more diagnoses with same ICD-9 Code |
|  | 279.x 495.0x, 495.1x, 495.2x, 495.3x, 495.4x, 495.5x, 495.6x, 495.7x, 495.9 x,518.3x 518.6x 530.19x 558.3x 716.2x,995.3x | 2 or more diagnoses with same ICD-9 Code |
| Serious local reactions | 682.3x 682.6x 682.9x 726.10x 726.19x 727.3x 729.5x 729.81x 999.39x | 1 or more diagnoses with same ICD-9 Code |
| Stevens-Johnson syndrome | 322.8x 323.8x 695.1x | 1 or more diagnoses with same ICD-9 Code |
| Urticaria | 708.0x 708.1x 708.9x | 1 or more diagnoses with same ICD-9 Code |

| **Asthma** | **ICD-9 Codes** | **Number of Required Diagnoses** |
| --- | --- | --- |
| Asthma | 493.xx V03.82 V06.6 v117.5 518.3x 518.81x 518.83x 518.84x 518.82x 519.11x 786.07x 495.8x 518.6x | 2 or more diagnoses within category |

| **Autoimmune** | **ICD-9 Codes** | **Number of Required Diagnoses** |
| --- | --- | --- |
| Inflammatory bowel disease | 555.xx, 556.xx, 558.3x 558.9x, 558.xx | 2 or more diagnoses with same ICD-9 Code |
| Other Autoimmune | 255.4x 258.1x 242.01x, 242.0x, 242.2x, 245.2x, 242.4x, 242.8x, 242.9x 704.0x 709.01x 694.4x 694.5x 694.9x  250.01 250.03 250.11 250.13 250.21 250.23 250.31 250.33 250.41 250.43 250.51 250.53 250.61 250.63 250.71 250.73 250.81 250.83 250.91 250.93  571.42x 571.49; 573.3x 576.1x 579.1x, 579.2x; 579.3x, 579.4x. 579.8x, 579.9x 571.6x 283.0x, V12.3 286.52 287.31x, 287.32x, 287.4x, 287.5x 273.2x 358.1x, 357.81x, 323.51; 323.52; 446.1x 775.2x 340.xx 358.0x 360.11x 360.12x 362.18x 363.2x 364.3x 376.12x 279.4x 386.0x 279.0x. 279.1x 279.2x 279.3x 279.5x 279.8x 279.9x 583.89x 583.9x 714.0x 714.2x  714.3x 390 – 398 701.0x 710.8x 710.9x 446.20 446.29 710.3x 710.4x  446.21 373.34x 695.4x 710.0x 710.1x 720.0x 720.9x 359.71 517.2x 728.11x 728.81x 714.1x 714.9x 710.2x 733.99 725.xx 696.0x 696.1x  099.3x 711.1x 711.90 135.xx 517.8x 321.4x 446.0x 446.4x 446.5x 446.7x 447.6x 447.8x 443.0x 136.1x 711.20 579.0x 516.31 697.1x 697.8x 697.9x  377.30x 377.31x 377.39 446.6x | 2 or more diagnoses with same ICD-9 Code |
| Other dermatitis autoimmune | 697.0x; 709.00; 709.09; 709.1x, 709.2x; 709.3x; 709.4x; 709.8x; 709.9x | 2 or more diagnoses with same ICD-9 Code |

| **Cardiovascular** | **ICD-9 Codes** | **Number of Required Diagnoses** |
| --- | --- | --- |
| Cardiovascular | 410.x 398.91; 402.01; 402.11; 402.91; 404.01; 404.03; 404.11; 404.13; 404.91; 404.93; 428.0; 428.1; 428.20; 428; 21; 428.22; 428.23; 428.30; 428.31; 428.32; 428.33; 428.40; 428.41; 428.42; 428.43 428.9x 272.2x 272.4x 401.x – 405.x 433.x 434.x 436.x 438.x 411.x | 1 or more diagnoses with same ICD-9 Code |

| **Development Delay** | **ICD-9 Codes** | **Number of Required Diagnoses** |
| --- | --- | --- |
| Language delay | 315.3X | 2 or more diagnoses within same category |
| Leaning & Cognitive disorder | 294.9x 331.83 315.0x 315.1x 315.2x | 2 or more diagnoses within same category |
| Global delay | 783.42, 315.5, 315.8, 315.9 | 2 or more diagnoses within same category |
| Motor delay | 315.4 | 1 or more diagnoses within same category |

| **Ear, nose and throat conditions** | **ICD-9 Codes** | **Number of Required Diagnoses** |
| --- | --- | --- |
| Ear, nose and throat conditions | 389.xx 744.xx 749.xx V41.2 V41.3 V49.85 380.3x 380.4x 380.8x 380.9x 380.22x | 1 or more diagnoses with same ICD-9 Code |

| **Endocrine** | **ICD-9 Codes** | **Number of Required Diagnoses** |
| --- | --- | --- |
| Endocrine disorders | 255.xx 249.xx 250.02x 250.10x 250.12x 250.20x 250.22x 250.30x 250.32x 250.40x 250.42x 250.50x 250.52x 250.60x 250.62x 250.70x 250.72x 250.80x 250.82x 250.90x 250.92x 252.xx 253.xx 254.xx 251.xx 258.xx 256.xx 257.xx 259.xx 246.xx 244.xx 243.xx 242.xx 240.xx 241.xx | 2 or more diagnoses within same category |

| **Gastrointestinal** | **ICD-9 Codes_kids** | **Number of Required Diagnoses** |
| --- | --- | --- |
| GERD | 530.81 | 2 or more diagnoses within same category |
| Diarrhea | 787.7x 787.91x | 2 or more diagnoses within same category |
| Constipation | 307.7x 787.6x, 564.0x | 2 or more diagnoses within same category |
| Upper GI diseases | 750.xx | 1 or more diagnoses with same ICD-9 Code |
| Upper GI motility | 307.53x 787.3x | 2 or more diagnoses with same ICD-9 Code |
|  | 787.0x | 2 or more diagnoses within same category |
| Functional disorders | 564.1x 564.2x 564.3x 564.4x 564.5x 564.6x 564.7x 564.9x 789.0x | 2 or more diagnoses within same category |
| Lower GI | 560.xx 578.xx 322.4x 323.4x | 1 or more diagnoses with same ICD-9 Code |
| Other disease of esophagus | 530.0x 530.1x 530.2x 530.3x 530.4x 530.5x 530.6x 530.7x 530.80 530.82 530.83 530.84 530.85 530.86 530.87 530.89 787.1x 787.2x | 2 or more diagnoses within same category |
| Other GI | 306.4x 577.xx 569.0x 569.3x 569.4x 579.xx 536.xx 537.xx 574.xx 575.xx 576.xx 571.xx 572.2x 572.3x 572.4x 572.8x 789.1x, 573.0x, 573.3x, 573.8x, 573.9x 789.7x 789.3x 789.4x 789.5x 789.6x 789.9x 789.2x  531.xx 532.xx 533.xx 534.xx 538.xx | 2 or more diagnoses within the same category |
|  | 070.4x 570.xx 573.4x 569.1 569.2 275.0x 278.xx 280.xx 783.1x 783.2x 783.3x 783.4x 783.5x 783.6x 783.9x V78.0 751.xx 756.6x 756.7x | 1 or more diagnoses with same ICD-9 Code |
|  | 793.6x 557.xx 558.xx | 2 or more diagnoses with same ICD-9 Code |

| **Genetic Conditions** | **ICD-9 Codes** | **Number of Required Diagnoses** |
| --- | --- | --- |
| Genetic Conditions | 758.7x 758.0x 330.8x 759.5x 759.6x 759.83x 758.1x 758.2x 758.3x 758.4x 758.5x 758.6x 758.8x 758.9x 759.81x 759.89x 759.81x 759.89x 237.7x 237.71x 237.72x | 2 or more diagnoses with same ICD-9 Code |

| **Genitourinary** | **ICD-9 Codes** | **Number of Required Diagnoses** |
| --- | --- | --- |
| Genital tract | 621.xx 608.xx | 1 or more diagnoses with same ICD-9 Code |
|  | 617.xx 626.xx 603.xx 605.xx 607.xx 600.xx 601.8x 601.9x 606.xx | 2 or more diagnoses with same ICD-9 Code |
| Renal disorders | 592.xx 594.xx, 753.xx | 1 or more diagnoses with same ICD-9 Code |
|  | 591.xx 593.xx 595.1X 595.2X 595.4X 595.8X 595.9X 596.xx 597.xx 599.xx 580.xx 581.xx 582.xx 583.xx 584.xx 585.xx 586.xx 587.xx 588.xx 589.xx 791.xx | 2 or more diagnoses with same ICD-9 Code |

| **Hematology** | **ICD-9 Codes** | **Number of Required Diagnoses** |
| --- | --- | --- |
| Hematology anemia | 282.xx 283.xx 284.xx 285.xx | 1 or more diagnosis within same category |
|  | 280.xx 281.xx | 2 or more diagnoses with same ICD-9 Code |
| Neoplasms | 140.xx 141.xx 142.xx 143.xx 144.xx 145.xx 146.xx 147.xx 148.xx 149.xx 150.xx 151.xx 152.xx 153.xx 154.xx 155.xx 156.xx 157.xx 158.xx 159.xx 160.xx 161.xx 162.xx 163.xx 164.xx 165.xx 170.xx 171.xx 172.xx 173.xx 174.xx 175.xx 176.xx 179.xx 180.xx 181.xx 182.xx 183.xx 184.xx 185.xx 186.xx 187.xx 188.xx 189.xx 190.xx 191.xx 192.xx 193.xx 194.xx 195.xx 196.xx 197.xx 198.xx 199.xx 200.xx 201.xx 202.xx 203.xx 204.xx 205.xx 206.xx 207.xx 208.xx 209.xx 210.xx 211.xx 212.xx 213.xx 214.xx 215.xx 216.xx 217.xx 218.xx 219.xx 220.xx 221.xx 222.xx 223.xx 224.xx 225.xx 226.xx 227.xx 228.xx 229.xx 230.xx 231.xx 232.xx 233.xx 234.xx 235.xx 236.xx 237.xx 238.xx 239.xx | 2 or more diagnoses with same ICD-9 Code |
| Other hematology | 286.xx 287.xx 288.xx 289.xx | 2 or more diagnoses with same ICD-9 Code |

| **Infection** | **ICD-9 Codes** | **Number of Required Diagnoses** |
| --- | --- | --- |
| Bacterial | 010.xx 011.xx 012.xx 013.xx 014.xx 015.xx 016.xx 017.xx 018.xx 020.xx 021.xx 022.xx 023.xx 024.xx 025.xx 026.xx 027.xx 030-041 031.xx 032.xx 033.xx 034.xx 035.xx 036.xx 037.xx 038.xx 039.xx 040.xx 041.xx 098.xx 325.xx 390.xx 391.xx 392.xx 073.xx 076.xx 590.xx | Counted as new infection if another dx > 30 days |
| Ear | 380.0x 380.14x 380.15x 380.16x 380.21x 380.23x, 380.3x, 380.4x, 380.8x, 380.9x 381.0x 381.1x 381.2x 381.3x 381.4x 381.5x 382.xx 383.0x 383.1x 383.2x 383.8x 383.9x 384.0x 384.1x 385.xx 386.0x 386.1x 386.2x 386.3x 387.xx 388.0x 388.1x 388.2x 388.3x 388.4x 388.5x 388.8x 388.9x | Counted as new infection if another dx > 14 days |
| Eye | 372.xx 373.0x 373.1x 373.4x 373.5x 373.6x 373.8x 373.9x 363.0 363.1 363.2 364.0 364.1 364.2 364.3 360.0 360.1, V49.85 | Counted as new infection if another dx > 7 days |
| Female Genitourinary | 614.xx 615.xx 616.xx | Counted as new infection if another dx > 30 days |
| Gastrointestinal | 001.xx 002.xx 003.xx 004.xx 005.xx 006.xx 007.xx 008.xx 009.xx 535.xx 540.xx 567.xx | Counted as new infection if another dx > 14 days |
| Genitourinary | 590.xx 595.xx 597.xx | Counted as new infection if another dx > 30 days |
| Lower Respiratory | 466.xx 490.xx 491.xx 770.0x | Counted as new infection if another dx > 30 days |
| Lymph | 289.1x 289.2x 289.3x 785.6x | Counted as new infection if another dx > 30 days |
| Mycoses | 117.9x 484.7x 711.6x 110.xx 111.xx 112.xx 113.xx 114.xx 115.xx 116.xx 117.xx 118.xx | Counted as new infection if another dx > 30 days |
| Other | 320.xx 321.xx 322.xx 323.xx 324.xx 326.xx 527.2x 527.3x 323.xx 324.xx 326.xx 771.xx 357.0x 383.xx 522.5x 522.6x 522.7x 601.xx 604.xx 422 420 421 424.9 | Counted as new infection if another dx > 30 days |
| Skin | 682.9x 686.8x 686.9x 078.0x 078.1x 680.xx 681.xx 682.xx 683.xx 684.xx 685.xx 686.xx | Counted as new infection if another dx > 14 days |
| Upper Respiratory | 465.8x 465.9x 460.xx 461.xx 462.xx 463.xx 464.xx 465.xx 472.xx 473.xx 476.xx | Counted as new infection if another dx > 21 days |
| Viral | 480.1x 480.2x 480.8x 488.01x 488.02x 488.09x 488.11x 488.12x 488.19x 488.81x 488.82x 488.89x 795.71x 042.xx 045.xx 046.xx 047.xx 048.xx 049.xx 050.xx 051.xx 052.xx 053.xx 054.xx 055.xx 056.xx 057.xx 058.xx 059.xx 055.2x 060.xx 061.xx 062.xx 063.xx 064.xx 065.xx 066.xx 070.xx 071.xx 072.xx 074.xx 075.xx 070-077 74.8 77.99 078.2-079 79.1 79.2 79.51 79.53 79.6 79.99 080-088 090-099 480-488 487.X 771 79.52 V04.82 V08 V65.44 | Counted as new infection if another dx > 30 days |

| **Injury** | **ICD-9 Codes** | **Number of Required Diagnoses** |
| --- | --- | --- |
| Accidental poisoning | 964.1x V82.5 E860.x E861.x E862.x E863.x E864.x E865.x E866.x E867.x E868.x E869.x | 1 or more diagnoses with same ICD-9 Code |
| Fracture | V15.51 V13.51 800.xx 801.xx 802.xx 803.xx 804.xx 805.xx 806.xx 807.xx 808.xx 809.xx 810.xx 811.xx 812.xx 813.xx 814.xx 815.xx 816.xx 817.xx 818.xx 819.xx 820.xx 821.xx 822.xx 823.xx 824.xx 825.xx 826.xx 827.xx 828.xx 829.xx | 1 or more diagnoses with same ICD-9 Code |
| Injury of head or neck | 959.0x 850.xx | 1 or more diagnoses with same ICD-9 Code |

| **Mental Health** | **ICD-9 Codes** | **Number of Required Diagnoses for kids** |
| --- | --- | --- |
| Disruptive impulse conduct disorders | 313.81, 312.xx | 2 or more diagnoses within same category |
| Attention deficit disorders | 314.XX | 2 or more diagnoses within same category |
| Anxiety disorder | 300.0x, 300.2X, 309.20, 309.21, 309.24, 309.81 | 2 or more diagnoses within same category |
| Depression | 296.2x, 296.3x,296.82, 298.0, 300.4X, 301.12, 311.XX, 309.0X, 309.1X, 309.28 | 2 or more diagnoses within same category |
| Adjustment disorders | 309.22, 309.23, 309.29, 309.3X, 309.4X, 309.82, 309.83, 309.89, 309.9X, 313.0X 313.1X 313.2X 313.3X 313.9X 313.82 313.83 313.89 | 2 or more diagnoses within same category |
| Tic Disorder | 307.2x | 2 or more diagnoses within same category |
| Other Mental Health | 296.0x, 296.1x, 296.4x, 296.5x, 296.6x 296.7x, 296.80, 296.81, 296.89, 296.9x 297.3x 298.1x 298.2x 298.3x 298.4x 298.8x 298.9x 300.5 300.6 300.7 300.8 301.0 301.11 301.13 300.3x 295.xx 297.1x 301.10x 301.20 301.21 301.3x 301.4x 301.5x 301.6x 301.7x 301.8x 301.9x, 316.xx 307.3x 307.53x 307.54 307.59 301.22 290.xx 780.93 331.0x 331.83x 310.8x 294.1x 307.10 307.50 307.51 307.52 780.97x E950 - E958 E980 - E988 | 2 or more diagnoses within same category |
|  | 291.xx 292.xx 303.xx 304.xx 305.00 305.01 305.02  305.20 305.21 305.22 305.30 305.31 305.32 305.40 305.41 305.42 305.50 305.51 305.52 305.60 305.61 305.62 305.70 305.71 305.72 305.80 305.81 305.82 305.90 305.91 305.92 | 1 or more diagnoses with same ICD-9 |

| **Metabolic** | **ICD-9 Codes** | **Number of Required Diagnoses** |
| --- | --- | --- |
| Fluid, electrolyte, and acid-base balance | 276.xx | 2 or more diagnoses with same ICD-9 Code |
| Overweight, obesity and other hyperalimentation | 278.xx | 2 or more diagnoses with same ICD-9 Code |

| **Musculoskeletal** | **ICD-9 Codes** | **Number of Required Diagnoses** |
| --- | --- | --- |
| Musculoskeletal | 754.xx 755.xx 756.0X 756.1X 756.2X 756.3X 756.4X 756.5X 756.8X 756.9X 737.3x 322.5x 323.5x 719.4x | 1 or more diagnoses with same ICD-9 Code |

| **Neurology** | **ICD-9 Codes** | **Number of Required Diagnoses** |
| --- | --- | --- |
| Cerebral palsy | 343.xx | 1 or more diagnoses with same ICD-9 Code |
| Other Disorders of Central Nervous System | 249.3x 250.3x 780.01x 251.0x 359.xx 346.xx 772.2x 430.xx 767.0x 852.xx 432.9x 325.X 341.xx 342.xx 344.xx, 348.xx 349.xx 741.xx 781.xx V48.X 783.xx, 786.90x 433.xx 434.xx 435.xx 322.3x 323.3x 357.0x 780.2x | 1 or more diagnoses with same ICD-9 Code |
| Epilepsy and recurrent seizures | 322.1x 323.1x 345.xx 780.3x | 1 or more diagnoses with same ICD-9 Code |
| Other Congenital Anomalies of Nervous System | 742.xx | 1 or more diagnoses with same ICD-9 Code |
| Abnormalities of Skull Face | 742.10x 754.00x 756.0x | 1 or more diagnoses with same ICD-9 Code |
| Hereditary and Degenerative Diseases of Central Nervous System | 330.xx 331.xx 333.xx 334.xx 335.xx 336.xx 337.xx 338.xx | 1 or more diagnoses with same ICD-9 Code |
|  | 339.xx | 2 or more diagnoses with same ICD-9 Code |
| Paroxysmal | 779.0x 780.31x 780.32x | 1 or more diagnoses with same ICD-9 Code |
|  | 780.33x 780.39x | 2 or more diagnoses with same ICD-9 Code |
| Disorders of Peripheral Nervous System | 350.xx 351.xx 352.xx 353.xx 767.6x 354.xx 355.xx 356.xx 357.xx 358.xx | 1 or more diagnoses with same ICD-9 Code |
| Headache | 307.81X 784.0X | 2 or more diagnoses with same ICD-9 Code |
| Other Neurology | 249.3x 250.3x 780.01x 251.0x 359.xx 346.x 772.2x 430.xx 767.0x 852.xx 432.9x 433.xx 434.xx 435.xx  322.3x 323.3x 357.0x 780.2x | 1 or more diagnoses with same ICD-9 Code |

| **Nutrition** | **ICD-9 Codes** | **Number of Required Diagnoses** |
| --- | --- | --- |
| Symptom concerning nutrition metabolism and development | 275.0x 278.xx 280.xx 783.1x 783.2x 783.3x 783.4x 783.5x 783.6x 783.9x V78.0 | 1 or more diagnoses with same ICD-9 Code |

| **Ophthalmology** | **ICD-9 Codes** | **Number of Required Diagnoses** |
| --- | --- | --- |
| Ophthalmology | 360.2X 360.3X 360.4X 360.5X 360.6X 360.8X 360.9X 361.X 362.1X 362.2X 362.3X 362.4X 362.5X 362.6X 362.7X 362.8X 362.9X 363.3X 363.4X 363.5X 363.6X 363.7X 363.8X 363.9X 364.4X 364.5X 364.6X 364.7X 364.8X 364.9X 743.xx | 1 or more diagnoses with same ICD-9 Code |
|  | 364.4x 366.0X 366.1X 366.3X 366.4X 366.8X 366.9X 367.x 368.x 369.x 374.0X 374.1X 374.2X 374.3X 374.4X 374.5X 377 378 379.5 | 2 or more diagnoses with same ICD-9 Code |
|  | 369.xx | 2 or more diagnoses within same category |

| **Other** | **ICD-9 Codes** | **Number of Required Diagnoses** |
| --- | --- | --- |
| Fever of Unknown | 780.60 780.61 | 1 or more diagnoses with same ICD-9 Code |
| Unspecified adverse effect of drug | 995.2x | 1 or more diagnoses with same ICD-9 Code |
| Other unspecified symptoms or syndromes (including masturbation) | 307.9 | 2 or more diagnoses with same ICD-9 Code |

| **Pulmonary** | **ICD-9 Codes** | **Number of Required Diagnoses** |
| --- | --- | --- |
| Pulmonary | 415.xx 519.xx 512.xx 518.xx | 1 or more diagnoses with same ICD-9 Code |
|  | 416.xx 417.xx 748.xx 490.xx 511.xx 514.xx 516.xx 277.0x | 2 or more diagnoses with same ICD-9 Code |
|  | 491.xx 492.xx 494.xx 496.xx | 2 or more diagnoses within same category |

| **Sleep** | **ICD-9 Codes** | **Number of Required Diagnoses** |
| --- | --- | --- |
| Organic sleep apnea | 327.1x 327.2x | 1 or more diagnoses with same ICD-9 Code |
| Dyssomnia | 780.5x 307.40x 307.42x 307.44x 307.46x 307.47x 307.49x 327.42x 327.8x V69.5 | 1 or more diagnoses with same ICD-9 Code |
